# Serum matrix metalloproteinase-9 as a potential biomarker for obstructive sleep apnea severity

**DOI:** 10.1007/s11325-025-03287-2

**Published:** 2025-03-21

**Authors:** So Yeon Kim, Hyunyee Yoon, Seung Ho Choi, Jaeyoung Cho

**Affiliations:** 1 Division of Pulmonary and Critical Care Medicine, Department of Internal Medicine, Seoul National University Hospital, 101 Daehak-ro, Jongno-gu, Seoul, 03080 Republic of Korea; 2 Protein Immunology Core Facility, Seoul National University Hospital Biomedical Research Institute Center for Medical Innovation, Seoul, Republic of Korea; 3 Department of Internal Medicine, Healthcare Research Institute, Healthcare System Gangnam Center, Seoul National University Hospital, Seoul, Republic of Korea; 4 Department of Internal Medicine, Seoul National University College of Medicine, Seoul, Republic of Korea

**Keywords:** Obstructive sleep apnea, Severity, Biomarker, Hypoxemia, Matrix metalloproteinase

## Abstract

**Purpose:**

We aimed to investigate the associations between serum matrix metalloproteinase (MMP)-2 and MMP-9 levels and obstructive sleep apnea (OSA) severity with a focus on nocturnal hypoxemia.

**Methods:**

The OSA patients (*n* = 105) were recruited from a prospective sleep apnea cohort after polysomnography, with 27 healthy volunteers as the controls. OSA severity was assessed via the apnea–hypopnea index (AHI) and percent night time with SpO_2_ < 90% (T90).

**Results:**

The serum MMP-9 levels were significantly higher in the OSA patients (AHI ≥ 5/h, 68.8 ± 44.9 ng/mL) than the controls (49.0 ± 18.6 ng/mL, *p* < 0.001). The MMP-2 levels showed no significant differences. When grouped into T90 quartiles, the MMP-9 levels were higher in the OSA patients in the highest quartile compared to those in the lowest quartile or the controls (90.6 ± 56.3 ng/mL vs. 56.9 ± 31.9 ng/mL, *p* = 0.022; 90.6 ± 56.3 ng/mL vs. 49.0 ± 18.6 ng/mL, *p* = 0.002, respectively). The MMP-9 levels correlated with T90 and the AHI (*r* = 0.36, *p* < 0.001; *r* = 0.35, *p* < 0.001, respectively). Multiple linear regression confirmed a significant association between MMP-9 and T90 after adjusting for body mass index, smoking status, and comorbidities (β = 0.53, *p* = 0.013). A similar association was observed for the AHI (β = 0.48, *p* = 0.019).

**Conclusion:**

We concluded that serum MMP-9 levels are independently associated with OSA severity, particularly with T90 and the AHI, which suggests that MMP-9 could be a biomarker for OSA severity.

**Supplementary Information:**

The online version contains supplementary material available at 10.1007/s11325-025-03287-2.

## Introduction

Obstructive sleep apnea (OSA) is a growing public health concern owing to its increasing prevalence, underdiagnosis, and substantial impact on health outcomes [1, 2]. OSA is associated with various comorbidities, including cardiovascular disease, metabolic syndrome, and neurocognitive impairments. Despite the importance of a timely diagnosis, the current gold standard for the diagnosis of OSA—overnight in-lab polysomnography (PSG)—is time-consuming, labor-intensive, expensive, and often inaccessible in resource-limited settings. This underscores the urgent need for alternative, accessible biomarkers to aid in the early detection and management of OSA.

Matrix metalloproteinases (MMPs), in particular, MMP-2 and MMP-9, have been investigated for their role as potential blood biomarkers for OSA, as they are related to the pathophysiological mechanisms of OSA, such as chronic intermittent hypoxia and systemic inflammation [3–5]. However, most such studies have been limited due to their small sample sizes. In addition, previous studies have primarily focused on the apnea–hypopnea index (AHI)—the frequency of apneas and hypopneas during sleep—as a metric of OSA severity. Although the AHI is widely used to indicate the severity of OSA, criticism of the index is increasing based on pathophysiologic grounds [6]: It fails to fully explain the symptoms of OSA and occasionally to predict disease outcomes [6, 7].

Given these limitations, in the present study we aimed to investigate whether serum MMP-2 and MMP-9 levels are independently associated with OSA severity by focusing on measures of nocturnal hypoxemia.

## Methods

### Study design and participants

Patients with OSA who participated in the Seoul National University Hospital (SNUH) Sleep Apnea Cohort (NCT04186078) between November 2019 and April 2023 were recruited for this cross-sectional study. The SNUH Sleep Apnea Cohort is an ongoing prospective cohort that enrolls patients aged 19 years or older who visit the outpatient clinic at SNUH with symptoms of sleep apnea, such as snoring, observed apnea, and daytime sleepiness, that require sleep studies for diagnosis. The present study included consecutive patients with OSA diagnosed with an AHI ≥ 5/h based on in-lab PSG and whose peripheral venous samples were collected during cohort enrollment. Patients with concomitant central sleep apnea were excluded from the study. The control participants were recruited from the Controls for Respiratory Diseases Cohort (NCT03120481), which enrolled healthy volunteers aged 19 years or older who had undergone health checkups at SNUH Healthcare System Gangnam Center in 2017 [8]. The inclusion criteria for the controls were available peripheral venous samples collected during health checkups; no previous history of hypertension, diabetes mellitus, dyslipidemia, coronary artery disease, stroke, chronic kidney or liver disease, cancer, respiratory diseases (e.g., chronic obstructive pulmonary disease [COPD], asthma, tuberculosis), allergic rhinitis, and autoimmune diseases; no significant chest radiographic abnormalities; a glycated hemoglobin (HbA1c) < 6.5%; and total CO_2_ < 27 mmol/L to exclude the presence of hypoventilation disorders. The participants in the control group were selected to match the age, sex, body mass index (BMI), and smoking status (never, former, or current) of the patients with OSA.

This study was approved by the Institutional Review Board of SNUH (H-2302-083-1404) and conducted in accordance with the tenets of the Declaration of Helsinki. All the participants provided their written informed consent.

### PSG and data collection for the case group

The participants enrolled in the SNUH Sleep Apnea Cohort underwent in-lab PSG at the Sleep Center of SNUH, which was conducted in line with the American Academy of Sleep Medicine (AASM) guidelines [9]. Apnea was defined as a complete or near complete (≥ 90%) cessation of airflow lasting at least 10 s. Hypopnea was identified as a reduction in the nasal pressure signal by at least 30% from the baseline for at least 10 s, accompanied by a 3% or greater drop in oxygen saturation (SpO_2_) from the baseline or arousal. In addition to the AHI and respiratory disturbance index (RDI), polysomnographic parameters, namely, the percent night time with SpO_2_ < 90% (T90) and mean and lowest SpO_2_, were recorded to assess the severity of hypoxemia.

Each patient with OSA enrolled in the SNUH Sleep Apnea Cohort completed surveys that addressed their demographics, smoking status, and comorbidities, as well as the questionnaires for the Epworth sleepiness scale (ESS) [10, 11] and Pittsburgh sleep quality index [12, 13]. Anthropometric measurements were assessed using bioelectrical impedance analysis (Inbody 970, Seoul, South Korea). The patients also underwent routine laboratory and pulmonary function tests. Peripheral venous samples were collected from all consenting patients during cohort enrollment.

### Data collection for the control group

The control participants completed surveys that queried their demographics, smoking status, and comorbidities. They provided peripheral venous samples on the date of the health checkups, and the results of the health checkups included routine laboratory and pulmonary function tests and bioelectrical impedance analysis.

### MMP measurement

The serum MMP-2 and MMP-9 levels were analyzed using ProcartaPlex™ Human, NHP, and Canine Mix & Match Panels (Invitrogen, Thermo Fisher Scientific, Waltham, MA, USA) in accordance with the manufacturer’s instructions. The reagents were prepared by mixing 20 mL of wash buffer concentrate (10×) with 180 mL of deionized water and subsequently stored at 2–8 °C for up to 6 months. The thawed serum samples were centrifuged at 1,000 × g for 10 min, then 25 µL of 1× Universal Assay Buffer and 25 µL of the samples were added to wells containing 50 µL of vortexed Capture Bead Mix. After incubation at room temperature for 2 h with shaking, the wells were washed twice. Subsequently, 25 µL of Biotinylated Detection Antibody Mix and 50 µL of Streptavidin-PE were added, each followed by incubation and washing. Finally, 120 µL of reading buffer was added to each well, which was incubated for 5 min, and the plate read. The data were acquired using a Bio-Plex 200 system analyzer (Bio-Rad, Hercules, CA, USA) while ensuring a minimum bead count of 50. The MMP levels were calculated using the Bio-Plex Manager™ program version 6.2 with 5PL curve fitting to account for the sample dilution factors. The laboratory staff was blinded to the patients’ data while analyzing the serum samples.

### Statistical analyses

The categorical variables were presented as counts and percentages and the continuous variables as means with standard deviations (SD). Any missing values related to the body composition parameters and the results of the routine laboratory and pulmonary function tests for the case group were imputed using multivariate imputation via the chained equations package (MICE, version 3.14.0) in R [14]. The severity of OSA was assessed by the AHI, RDI, and T90, which were considered continuous or categorized. The AHI or RDI was classified by the cutoffs 5, 15, and 30/h as mild, moderate, and severe OSA, respectively [7, 15]. T90 was categorized as a quartile. T90 was also categorized as suggested in previous studies of Spanish [16] and French cohorts [17], respectively. The continuous variables were compared using Student’s *t*-tests or one-way analysis of variance with Tukey’s honest significance test. For the categorical variables, comparisons were undertaken using either χ^2^ or Fisher’s exact tests. Pearson’s correlation coefficient was used to determine the correlations between the various sleep parameters and MMP-9 levels in the patients with OSA.

In patients with OSA, multiple linear regression was applied to assess the relationship between the severity of OSA (i.e., the AHI and T90) and the serum MMP-9 levels after adjusting for potential confounders—both known confounders, such as BMI and smoking status, identified in previous research [18, 19] and variables selected by the least absolute shrinkage and selection operator (Lasso) method (R package: glmmLasso, version 1.6.2) [20]. The use of the Lasso method shrinks some variable coefficients and sets others to zero in an attempt to maintain appropriate variables for subset selection. We fitted two multivariable models separately for each severity index. All the comparisons were two-sided, and *p* < 0.05 was considered statistically significant. All the analyses were performed using R version 4.4.2.

## Results

### Baseline characteristics of the study participants

Four of the 109 patients with OSA who were enrolled in the SNUH Sleep Apnea Cohort and provided blood samples were excluded from the study due to the presence of concomitant central sleep apnea. Of the 139 participants enrolled in the Controls for Respiratory Diseases Cohort who met the study inclusion criteria, 27 were selected as their age, sex, BMI, and smoking status matched those of the patients with OSA. As shown in Table 1, there were no significant differences in age, sex, and smoking status between the case and control groups. However, the mean BMI of the patients with OSA was significantly higher: Based on the definition of obesity of the World Health Organization and the Asian-Pacific guidelines [21], 77% of the patients with OSA were obese compared to 37% of the controls. Similarly, the mean waist circumference and fat mass were greater in the case group than the controls.

**Table 1:** Baseline characteristics

Characteristic	OSA (*n* = 105)	Control (*n* = 27)	*p*
Age, years	58.2 ± 10.8	56.0 ± 8.9	0.340
Male sex	78 (74.3)	20 (74.1)	> 0.999
Smoking status			0.880
Never	43 (41.0)	10 (37.0)	
Former	46 (43.8)	12 (44.4)	
Current	16 (15.2)	5 (18.5)	
Heavy drinker^*^	0 (0.0)	0 (0.0)	1.000
BMI, kg/m^2^	27.8 ± 4.0	25.6 ± 3.2	0.007
BMI, kg/m^2^			< 0.001
18.5–22.9	7 (6.7)	3 (11.1)	
23–24.9	17 (16.2)	14 (51.9)	
≥ 25	81 (77.1)	10 (37.0)	
Waist circumference, cm	95.8 ± 10.1	89.2 ± 8.1	0.002
Skeletal muscle mass, kg	29.6 ± 6.0	29.3 ± 6.1	0.819
Fat mass, kg	23.7 ± 8.5	20.0 ± 6.7	0.034
Percentage body fat, %	30.6 ± 7.7	27.5 ± 7.4	0.064
Waist-to-hip ratio	0.9 ± 0.1	0.9 ± 0.1	0.211
Systolic BP, mmHg	136 ± 14	121 ± 14	< 0.001
Diastolic BP, mmHg	81 ± 12	79 ± 9	0.441
WBC, ×10^3^/µL	6.6 ± 2.0	5.3 ± 1.4	0.002
Eosinophils, ×10^3^/µL	2.7 ± 2.1	3.1 ± 2.3	0.310
Hemoglobin, g/dL	14.5 ± 1.8	14.5 ± 2.0	0.981
Platelets, ×10^3^/µL	238.1 ± 62.7	240.6 ± 61.7	0.854
Glucose, mg/dL	113.6 ± 28.3	99.1 ± 10.8	< 0.001
Protein, g/dL	7.2 ± 0.4	7.1 ± 0.4	0.221
Albumin, g/dL	4.5 ± 0.3	4.4 ± 0.2	0.211
AST, IU/L	27.4 ± 27.5	24.9 ± 8.7	0.442
ALT, IU/L	28.8 ± 16.4	28.5 ± 20.7	0.944
BUN, mg/dL	16.9 ± 6.1	14.3 ± 3.7	0.006
Creatinine, mg/dL	0.9 ± 0.3	0.9 ± 0.2	0.186
Total CO_2_, mmol/L	28.8 ± 3.1	24.6 ± 1.6	< 0.001
HbA1c, %	5.9 ± 0.7	5.6 ± 0.4	0.003
Total cholesterol, mg/dL	190 ± 133	193 ± 32	0.875
Triglycerides, mg/dL	137 ± 81	134 ± 90	0.877
HDL cholesterol, mg/dL	49 ± 13	52 ± 12	0.312
LDL cholesterol, mg/dL	107 ± 35	122 ± 30	0.039
FVC, L	3.7 ± 1.0	3.8 ± 0.9	0.577
FVC, %	93.5 ± 16.1	94.8 ± 13.8	0.697
FEV_1_, L	2.7 ± 0.8	3.0 ± 0.7	0.068
FEV_1_, %	96.8 ± 19.9	105.5 ± 16.2	0.037
FEV_1_/FVC, %	74.5 ± 8.4	81.2 ± 4.5	< 0.001

Values are presented as mean ± SD or number (%)

^*^Heavy drinkers were defined as individuals who drank at least twice a week, and the average amount of alcohol consumed ≥ 7 drinks at a time for men and ≥ 5 drinks for women

Abbreviations: ALT, alanine transaminase; AST, aspartate transaminase; BMI, body mass index; BP, blood pressure; BUN, blood urea nitrogen; FEV_1_, forced expiratory volume in 1 s; FVC, forced vital capacity; HbA1c, glycated hemoglobin; HDL, high-density lipoprotein; LDL, low-density lipoprotein; WBC, white blood cells

Only one of the control participants reported symptoms of insomnia. Table 2 delineates the clinical characteristics of the 105 patients with OSA. Among them, 14 (13.3%) had mild OSA, 32 (30.5%) had moderate OSA, and 59 (56.2%) had severe OSA. The mean T90 was 12.4% ± 22.4%, and the lowest SpO_2_ was 77.3% ± 10.6%. As expected, these patients frequently presented with cardiometabolic comorbidities. The mean ESS score was 7.3 ± 4.4.

**Table 2:** Clinical characteristics of patients with OSA

Characteristics	OSA (*n* = 105)
Comorbidities	
Hypertension	69 (65.7)
Coronary artery disease	17 (16.2)
Stroke	11 (10.5)
Diabetes mellitus	33 (31.4)
Dyslipidemia	64 (61.0)
Gastroesophageal reflux disease	45 (42.9)
Chronic kidney disease	13 (12.4)
Chronic obstructive pulmonary disease	11 (10.5)
Asthma	9 (8.6)
Cancer	14 (13.3)
Restless legs syndrome	6 (5.7)
Questionnaires	
STOP-Bang questionnaire	5.1 ± 1.3
Epworth sleepiness scale	7.3 ± 4.4
Pittsburgh sleep quality index	7.9 ± 3.7
Insomnia severity index	8.9 ± 5.4
Beck depression inventory	11.2 ± 8.8
Polysomnography	
AHI, /h	37.5 ± 22.1
AHI, /h	
5–14.9	14 (13.3)
15–29.9	32 (30.5)
≥ 30	59 (56.2)
Supine AHI, /h	52.2 ± 27.2
Nonsupine AHI, /h	21.5 ± 23.5
REM AHI, /h	39.8 ± 23.0
NREM AHI, /h	34.9 ± 23.1
Isolated REM OSA^*^	4 (3.8)
RDI, /h	38.0 ± 22.0
T90, %	12.4 ± 22.4
Mean SpO_2_, %	91.5 ± 4.3
Lowest SpO_2_, %	77.3 ± 10.6

Values are presented as mean ± SD or number (%)

^*^Isolated REM OSA was defined as an overall AHI ≥ 5/h, a REM AHI/NREM AHI ratio ≥ 2, a NREM AHI < 5/h, a REM AHI > 5/h, and REM sleep ≥ 30 min

Abbreviations: AHI, apnea–hypopnea index; NREM, nonrapid eye movement; OSA, obstructive sleep apnea; RDI, respiratory disturbance index; REM, rapid eye movement; SpO_2_, oxygen saturation; STOP-Bang questionnaire, Snoring, Tiredness, Observed apnea, high blood Pressure-Body mass index, Age, Neck circumference, and Gender questionnaire; T90, percent night time with oxygen saturation < 90%

### Serum MMP levels

**Fig. 1:**
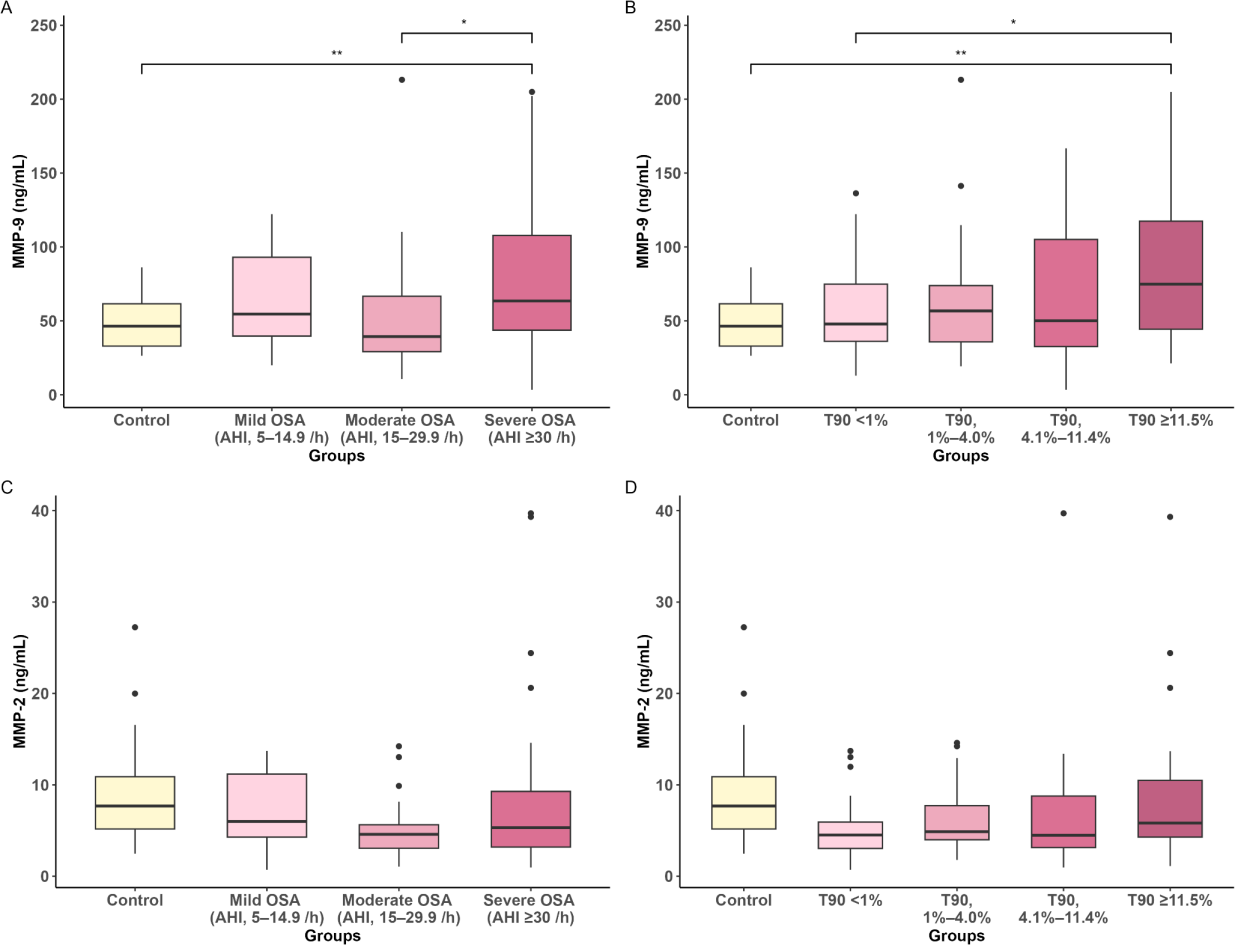
Serum MMP-9 (**A**, **B**) and MMP-2 (**C**, **D**) levels according to the AHI and T90. The groups were compared by one-way analysis of variance followed by Tukey’s honest significance test. *p < 0.05; **p < 0.01. AHI, apnea–hypopnea index; MMP-9, matrix metalloproteinase-9; OSA, obstructive sleep apnea; T90, percent night time with oxygen saturation < 90%

The serum MMP-9 levels were significantly higher in the patients with OSA than in the controls (68.8 ± 44.9 ng/mL vs. 49.0 ± 18.6 ng/mL, *p* < 0.001, respectively). However, the serum MMP-2 levels were not significantly different between the case and control groups (6.7 ± 6.2 ng/mL vs. 9.0 ± 5.7 ng/mL, *p* = 0.084, respectively). Figure 1 shows the relationships between the indices of OSA severity and the serum MMP levels. The serum MMP-9 levels were higher in the patients with severe OSA than in those with moderate OSA and the controls (78.8 ± 48.0 ng/mL vs. 51.3 ± 37.8 ng/mL, *p* = 0.011; 78.8 ± 48.0 ng/mL vs. 49.0 ± 18.6, *p* = 0.009, respectively; Fig. 1A). The serum MMP-9 levels showed similar results for the RDI (Supplementary Figure S1A). When the patients with OSA were grouped into the T90 quartiles (< 1%, 1–4.0%, 4.1–11.4%, ≥ 11.5%), the serum MMP-9 levels of the patients with OSA in the highest T90 quartile were higher than those in the lowest T90 quartile and the controls (90.6 ± 56.3 ng/mL vs. 56.9 ± 31.9 ng/mL, *p* = 0.022; 90.6 ± 56.3 ng/mL vs. 49.0 ± 18.6 ng/mL, *p* = 0.002, respectively; Fig. 1B). The baseline characteristics of the patients with OSA according to the T90 quartiles are shown in Supplementary Table S1. The serum MMP-9 levels according to the different T90 categories showed similar trends when T90 was categorized into tertiles (< 1.2%, 1.2–12%, > 12%), as in a Spanish cohort (Supplementary Figure S2A) [16], and quartiles (< 0.01%, 0.01–2.1%, 2.1–13%, ≥ 13%), as per a French cohort (Supplementary Figure S2B) [17]. The serum MMP-2 levels did not differ according to the AHI (Fig. 1C), RDI (Supplementary Figure S1B), and T90 (Fig. 1D, Supplementary Figures S2C and D). In addition, the serum MMP-9 and MMP-2 levels did not differ according to the Snoring, Tiredness, Observed apnea, high blood Pressure-Body mass index, Age, Neck circumference, and Gender (STOP-Bang) questionnaire scores (< 5 vs. ≥5; Supplementary Figure S3A and S3C) or ESS scores (< 10 vs. ≥10; Supplementary Figure S3B and S3D) in patients with OSA.

**Fig. 2:**
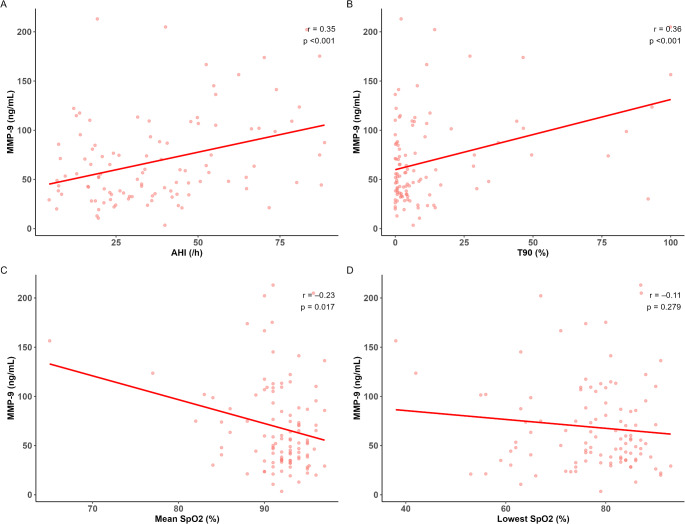
Correlation between the serum MMP-9 levels and several polysomnographic parameters in patients with OSA. (**A**) Apnea–hypopnea index (AHI). (**B**) Percent night time with oxygen saturation < 90% (T90). (**C**) Mean oxygen saturation (SpO_2_). (**D**) Lowest SpO_2_. MMP-9, matrix metalloproteinase-9; OSA, obstructive sleep apnea

The serum levels of MMP-9 were significantly positively correlated with the AHI and T90 in the patients with OSA (*r* = 0.35, *p* < 0.001, Fig. 2A; *r* = 0.36, *p* < 0.001, respectively, Fig. 2B) but negatively correlated with the mean SpO_2_ and lowest SpO_2_ (*r* = − 0.23, *p* = 0.017, Fig. 2C; *r* = − 0.11, *p* = 0.279, Fig. 2D, respectively). Supplementary Figure S4 shows the correlation coefficients between AHI or T90 and MMP-9 across comorbidity subgroups. Two multiple linear regression models were fitted separately for each severity index, T90 and AHI, as these indices were positively correlated (*r* = 0.49, *p* < 0.001). In the multiple linear regression, the association between the serum MMP-9 levels and T90 was statistically significant after adjusting for BMI, smoking status, and comorbidities such as hypertension, diabetes mellitus, COPD, and asthma in the patients with OSA (β = 0.53, standard error [SE] = 0.21, *p* = 0.013; Table 3). The association between the serum MMP-9 levels and the AHI showed similar findings (β = 0.48, SE = 0.20, *p* = 0.019; Table 3).

**Table 3:** Multiple linear regression of the serum MMP-9 levels in the patients with OSA

Variable	Model 1^*^		Model 2^†^	
	β (SE)	*p*	β (SE)	*p*
T90	0.53 (0.21)	0.013		
AHI			0.48 (0.20)	0.019
BMI	–1.13 (1.19)	0.343	–0.73 (1.15)	0.526
Smoking status (vs. never-smoker)				
Former	0.54 (8.89)	0.952	–1.61 (8.95)	0.857
Current	–21.37 (12.00)	0.078	–19.99 (12.02)	0.100
Hypertension	9.68 (8.81)	0.274	9.26 (8.90)	0.301
Diabetes mellitus	10.89 (9.22)	0.241	12.58 (9.18)	0.174
COPD	27.15 (14.74)	0.069	29.28 (14.65)	0.048
Asthma	21.29 (16.37)	0.197	19.80 (16.43)	0.231

Two multiple linear regression models were fitted separately for each severity index. ^*^Model 1 shows the association between serum MMP-9 levels and T90, adjusting for BMI, smoking status, and comorbidities, whereas ^†^Model 2 shows the association between serum MMP-9 levels and AHI

Abbreviations: AHI, apnea–hypopnea index; BMI, body mass index; COPD, chronic obstructive pulmonary disease; MMP-9, matrix metalloproteinase-9; OSA, obstructive sleep apnea; T90, percent night time with oxygen saturation < 90%

## Discussion

In the present study, we explored the relationship between serum MMP levels and the severity of OSA, with a particular focus on MMP-9. Our findings showed a significant association between serum MMP-9 levels and the severity of OSA, as measured by T90 and the AHI. The serum MMP-9 levels were significantly higher in the patients with severe OSA and were associated with T90 or the AHI after adjusting for potential confounders such as BMI, smoking status, and comorbidities including hypertension, diabetes mellitus, COPD, and asthma.

MMPs are a group of zinc-dependent proteinases, which are involved in the degradation and remodeling of the extracellular matrix. Of more than 20 different types of MMPs, MMP-9 has been implicated in various pathological processes, including inflammation, tissue injury, repair processes, and tumor progression [3–5]. MMP-9 is synthesized and released as inactive zymogen by inflammatory cells, fibroblasts, and the endothelium. It is activated extracellularly via proteolysis by different enzymes, such as plasminogen and tissue plasminogen activator, as well as MMPs themselves or via reactive oxygen species (ROS) directly [5]. Repeated episodes of partial or complete obstruction of the upper airway during sleep leading to chronic intermittent hypoxia is the pathophysiologic hallmark of OSA. ROS produced during chronic intermittent hypoxia can activate MMP-9 [5]. In addition, the activation of nuclear factor kappa B (NF-kB) in response to ROS leads to the upregulation of MMP-9 [22]. The overexpression and activation of MMP-9 by ROS and NF-kB may act as molecular mechanisms to explain the links between chronic intermittent hypoxia, inflammation, and endothelial dysfunction, all of which can lead to cardiovascular disease in patients with OSA [5]. MMP-9 is thus suggested as a biomarker candidate for OSA severity based on its pathophysiology.

The serum and plasma levels of MMP-9 in patients with OSA have been evaluated in previous studies [3–5, 23]. Although significant associations between MMP levels and severe OSA defined by the AHI were not found in some such studies [24–26], most studies reported increased MMP-9 levels, especially in severe OSA, and significant correlations between MMP-9 levels and the severity of OSA [23, 27, 28]. A meta-analysis showed that peripheral levels of MMP-9 were higher in patients with OSA compared to controls, and this increase was related to OSA severity [4]. However, only a few studies have examined the association between MMP-9 levels and measures of hypoxemia, including T90 [23, 24, 29]. The AHI is the most used metric for diagnosing OSA and assessing its severity. However, while the AHI is defined as the frequency of respiratory events, it frequently fails to reflect the clinical and physiological impacts of OSA. It further has a limited predictive ability for OSA symptoms, quality of life, and cardiovascular outcomes [6, 30]. As a result, alternative metrics for assessing disease severity have been proposed, and measures of hypoxemia, including T90, have shown better associations with adverse health outcomes, including cardiovascular events and mortality, compared to the frequency of respiratory events [31, 32]. We therefore comprehensively evaluated the associations between the serum levels of MMP-9 and MMP-2 and the metrics of event frequency (AHI and RDI) and nocturnal hypoxemia (T90, mean SpO_2_, and lowest SpO_2_). We categorized T90 as a quartile in this study. In addition, T90 was categorized as suggested in previous studies involving Spanish [16] and French cohorts [17], respectively. The associations between the serum MMP-9 levels and the different categories of T90 were reproducible. The patients with OSA in the highest category of T90—11.5% or more in our study, more than 12% in the Spanish cohort, and 13% or more in the French cohort—showed the highest levels of serum MMP-9.

MMP-9 serves as a common denominator in OSA, hypertension, coronary artery disease, and stroke, reflecting its role in inflammation, extracellular matrix remodeling, and vascular dysfunction [3]. Moreover, MMP-9 polymorphisms have been associated with an increased risk of developing type 2 diabetes [33]. Patients with COPD or asthma may experience hypoxia, which can alter their MMP-9 levels. Therefore, we performed correlation analyses in various comorbidity subgroups in Supplementary Figure S4; however, the small sample size limits the interpretation of the findings. Further investigation is needed to clarify the specific role of MMP-9 in OSA, particularly in the subgroups with stroke, coronary artery disease, or COPD.

To control for several factors that could affect MMP-9 levels during multiple linear regression, we adjusted for potential confounders, including known confounders such as BMI and smoking status [18, 19], identified in previous research, as well as those selected using the Lasso method in patients with OSA. All the confounders selected via the Lasso method— hypertension, diabetes mellitus, COPD, and asthma—are pathophysiologically related to MMP-9. After adjusting for these confounders, we showed that serum MMP-9 levels were independently associated with T90 or the AHI.

In this study, serum MMP-2 levels were not associated with the severity indices of OSA. Research on the relationship between MMP-2 and OSA severity is conflicting. One study reported that the serum MMP-2 levels were significantly lower in the patients with severe OSA (defined as an RDI > 30/h) compared to those with an RDI < 30/h [26]. Another study showed no association between serum MMP-2 levels and OSA severity defined by the AHI [24]. Hopps et al. reported increased plasma MMP-2 levels in patients with OSA; however, these levels were not higher in the patients with severe OSA (defined as an AHI > 30/h) compared to those with mild OSA [28]. A recent study showed that, after adjusting for age, sex, BMI, and cardiovascular disease, serum MMP-2 activity measured by gelatin zymography was associated with the AHI and oxygen desaturation index, which was defined as the frequency of oxygen desaturation drops by at least 3% [34]. However, correlations between serum MMP-2 activity and other measures of nocturnal hypoxemia, such as T90, mean SpO_2_, and lowest SpO_2_, were not shown in the study. This could be related to the potential impact of outliers in serum MMP-2 levels and the small sample size, which may have limited the statistical power to detect such an association.

A major limitation of this study was that the healthy control participants did not undergo PSG due to the retrospective nature of the control cohort. Questionnaires such as the STOP-Bang and the ESS were also not administered to the control participants. To minimize the risk of the presence of undiagnosed OSA in the healthy volunteers, we excluded participants with comorbidities that are frequently accompanied by sleep apnea, such as cardiometabolic diseases (e.g., hypertension, diabetes mellitus) and respiratory diseases (e.g., COPD, asthma). We also excluded those with a total CO_2_ of 27 mmol/L or more to eliminate the presence of hypoventilation disorders. However, it is still possible that patients with mild OSA without comorbidities were included in the control group.

Additional limitations are as follows: First, there were differences in BMI between the patients with OSA and the controls despite efforts to match them. Notwithstanding, the other variables (i.e., age, sex, smoking status) were well matched. Second, the activity of MMP-9 was not examined. The immunoassay technique used in this study measures the protein concentration based on the antigen–antibody reaction, whereas gelatin zymography assesses proteolytic activity, which may have been more biologically relevant [5]. Third, the treatment response data for the serum MMP-9 levels were absent. Previous studies have reported decreased levels and activity of MMP-9 following short-term treatment with continuous positive airway pressure [23], although this beneficial effect was not sustained over the long term (i.e., more than five years) [35]. Fourth, due to technical issues at the sleep center, hypoxic burden [36] or oxygen desaturation index was not analyzed in this study, although these metrics are associated with comorbid cardiovascular risk.

## Conclusion

In this study, we showed that serum MMP-9 levels were independently associated with the severity indices T90 (as a measure of nocturnal hypoxemia) and the AHI after adjusting for confounding comorbidities in the patients with OSA. These findings suggest that MMP-9 has potential as a biomarker for assessing OSA severity, although further large-scale prospective studies are needed to confirm its predictive performance.

## Electronic supplementary material

Below is the link to the electronic supplementary material.

Supplementary Material 1

## Data Availability

The data that support the findings of this study are available from the corresponding author upon reasonable request.
