# Supplementary material for: Serum matrix metalloproteinase-9 as a potential biomarker for obstructive sleep apnea severity
Source: Sleep Breath. 2025 Mar 21;29(2):134. doi: 10.1007/s11325-025-03287-2 (PMC11928356; doi:10.1007/s11325-025-03287-2)
Supplement: Supplementary file 1 — Supplementary Material 1 [file 11325_2025_3287_MOESM1_ESM.docx]

**Serum matrix metalloproteinase-9 as a potential biomarker for obstructive sleep apnea severity**

So Yeon Kim^1^, Hyunyee Yoon^2^, Seung Ho Choi^3^, Jaeyoung Cho^1,4,*^

^1^Division of Pulmonary and Critical Care Medicine, Department of Internal Medicine, Seoul National University Hospital, Seoul, Republic of Korea

^2^Protein Immunology Core Facility, Seoul National University Hospital Biomedical Research Institute Center for Medical Innovation, Seoul, Republic of Korea

^3^Department of Internal Medicine, Healthcare Research Institute, Healthcare System Gangnam Center, Seoul National University Hospital, Seoul, Republic of Korea

^4^Department of Internal Medicine, Seoul National University College of Medicine, Seoul, Republic of Korea

**Supplementary Material**

**TABLE S1** Baseline characteristics of patients with OSA according to T90

| **Characteristic** | **T90** | | | | ***P*** |
| --- | --- | --- | --- | --- | --- |
|  | **<1%**  **(n = 28)** | **1%–4.0%**  **(n = 27)** | **4.1%–11.4%**  **(n = 25)** | **≥11.5%**  **(n = 25)** |  |
| Age, years | 53.1 ± 12.8 | 61.0 ± 7.2 | 59.1 ± 8.0 | 59.9 ± 12.5 | 0.030 |
| Male sex | 23 (82.1) | 20 (74.1) | 17 (68.0) | 18 (72.0) | 0.684 |
| Smoking status |  |  |  |  | 0.593 |
| Never | 11 (39.3) | 12 (44.4) | 12 (48.0) | 8 (32.0) |  |
| Former | 10 (35.7) | 13 (48.1) | 10 (40.0) | 13 (52.0) |  |
| Current | 7 (25.0) | 2 (7.4) | 3 (12.0) | 4 (16.0) |  |
| Heavy drinker^*^ | 0 (0.0) | 0 (0.0) | 0 (0.0) | 0 (0.0) | 1.000 |
| Body composition parameters | | | | | |
| BMI, kg/m^2^ | 26.3 ± 3.0 | 27.0 ± 2.9 | 28.2 ± 3.6 | 30.1 ± 5.2 | 0.002 |
| BMI, kg/m^2^ |  |  |  |  | 0.144 |
| 18.5–22.9 | 4 (14.3) | 1 (3.7) | 0 (0.0) | 2 (8.0) |  |
| 23–24.9 | 6 (21.4) | 6 (22.2) | 4 (16.0) | 1 (4.0) |  |
| ≥25 | 18 (64.3) | 20 (74.1) | 21 (84.0) | 22 (88.0) |  |
| Neck circumference, cm | 38.9 ± 3.6 | 38.8 ± 3.9 | 40.1 ± 3.8 | 41.8 ± 4.9 | 0.031 |
| Waist circumference, cm | 91.5 ± 6.3 | 93.3 ± 9.1 | 96.9 ± 8.4 | 102.1 ± 12.7 | <0.001 |
| Skeletal muscle mass, kg | 29.8 ± 5.5 | 29.6 ± 6.5 | 29.2 ± 5.3 | 29.5 ± 6.8 | 0.99 |
| Fat mass, kg | 20.3 ± 6.2 | 21.9 ± 6.5 | 24.2 ± 6.6 | 29.1 ± 11.5 | 0.001 |
| Percentage body fat, % | 27.4 ± 7.6 | 29.6 ± 6.5 | 31.8 ± 7.4 | 33.9 ± 8.1 | 0.012 |
| Waist-to-hip ratio | 0.9 ± 0.0 | 0.9 ± 0.1 | 0.9 ± 0.1 | 1.0 ± 0.1 | 0.007 |
| Systolic BP, mmHg | 136 ± 16 | 132 ± 10 | 134 ± 13 | 143 ± 14 | 0.041 |
| Diastolic BP, mmHg | 84 ± 12 | 79 ± 7 | 79 ± 15 | 84 ± 12 | 0.280 |
| Laboratory data | | | | | |
| WBC, ×10^3^/μL | 6.5 ± 1.7 | 6.1 ± 1.8 | 6.2 ± 1.8 | 7.7 ± 2.2 | 0.015 |
| Eosinophils, ×10^3^/ μL | 2.7 ± 1.9 | 3.2 ± 2.7 | 2.2 ± 2.0 | 2.6 ± 1.7 | 0.414 |
| Hemoglobin, g/dL | 14.6 ± 1.5 | 14.6 ± 1.9 | 14.3 ± 2.0 | 14.6 ± 1.9 | 0.88 |
| Platelets, ×10^3^/μL | 243.2 ± 61.3 | 234.8 ± 52.8 | 240.2 ± 75.9 | 233.9 ± 63.0 | 0.942 |
| Glucose, mg/dL | 105.5 ± 12.3 | 114.0 ± 34.1 | 114.2 ± 29.6 | 121.4 ± 32.1 | 0.242 |
| Protein, g/dL | 7.1 ± 0.4 | 7.2 ± 0.4 | 7.0 ± 0.4 | 7.3 ± 0.5 | 0.156 |
| Albumin, g/dL | 4.5 ± 0.4 | 4.5 ± 0.3 | 4.3 ± 0.3 | 4.5 ± 0.4 | 0.145 |
| AST, IU/L | 34.7 ± 51.4 | 25.0 ± 8.4 | 22.2 ± 5.6 | 26.9 ± 9.9 | 0.384 |
| ALT, IU/L | 30.9 ± 18.2 | 31.6 ± 18.0 | 23.5 ± 9.7 | 28.8 ± 17.6 | 0.285 |
| BUN, mg/dL | 15.0 ± 3.1 | 17.1 ± 5.9 | 18.7 ± 8.4 | 17.1 ± 5.6 | 0.166 |
| Creatinine, mg/dL | 0.9 ± 0.2 | 0.9 ± 0.2 | 1.0 ± 0.5 | 0.9 ± 0.3 | 0.716 |
| Total CO_2_, mmol/L | 27.9 ± 2.7 | 29.8 ± 2.9 | 28.2 ± 3.5 | 29.3 ± 3.1 | 0.077 |
| HbA1c, % | 5.6 ± 0.3 | 5.8 ± 0.7 | 5.9 ± 0.8 | 6.4 ± 0.9 | 0.003 |
| Total cholesterol, mg/dL | 186 ± 42 | 226 ± 254 | 184 ± 39 | 164 ± 36 | 0.389 |
| Triglycerides, mg/dL | 152 ± 106 | 129 ± 64 | 127 ± 59 | 138 ± 87 | 0.684 |
| HDL cholesterol, mg/dL | 52 ± 13 | 49 ± 11 | 44 ± 13 | 49 ± 14 | 0.213 |
| LDL cholesterol, mg/dL | 111 ± 36 | 104 ± 34 | 119 ± 39 | 94 ± 30 | 0.074 |
| Pulmonary function test | | | | | |
| FVC, L | 4.1 ± 1.1 | 3.8 ± 0.8 | 3.4 ± 0.7 | 3.2 ± 1.1 | 0.005 |
| FVC, % | 97.5 ± 13.9 | 97.6 ± 14.4 | 91.8 ± 14.8 | 86.1 ± 19.1 | 0.026 |
| FEV_1_, L | 3.1 ± 0.8 | 2.8 ± 0.7 | 2.6 ± 0.6 | 2.4 ± 0.9 | 0.003 |
| FEV_1_, % | 101.0 ± 13.7 | 102.2 ± 18.9 | 94.9 ± 16.8 | 88.1 ± 26.4 | 0.038 |
| FEV_1_/FVC, % | 76.1 ± 8.1 | 74.7 ± 8.6 | 74.6 ± 6.3 | 72.2 ± 10.2 | 0.412 |
| Comorbidities | | | | | |
| Hypertension | 17 (60.7) | 15 (55.6) | 16 (64.0) | 21 (84.0) | 0.152 |
| Coronary artery disease | 2 (7.1) | 3 (11.1) | 3 (12.0) | 9 (36.0) | 0.039 |
| Stroke | 1 (3.6) | 3 (11.1) | 6 (24.0) | 1 (4.0) | 0.081 |
| Diabetes mellitus | 3 (10.7) | 9 (33.3) | 6 (24.0) | 15 (60.0) | 0.001 |
| Dyslipidemia | 17 (60.7) | 13 (48.1) | 14 (56.0) | 20 (80.0) | 0.115 |
| Gastroesophageal reflux disease | 14 (50.0) | 12 (44.4) | 9 (36.0) | 10 (40.0) | 0.759 |
| Chronic kidney disease | 2 (7.1) | 1 (3.7) | 5 (20.0) | 5 (20.0) | 0.145 |
| COPD | 2 (7.1) | 2 (7.4) | 1 (4.0) | 6 (24.0) | 0.150 |
| Asthma | 1 (3.6) | 1 (3.7) | 3 (12.0) | 4 (16.0) | 0.33 |
| Cancer | 2 (7.1) | 1 (3.7) | 6 (24.0) | 5 (20.0) | 0.094 |
| Restless legs syndrome | 2 (7.1) | 2 (7.4) | 1 (4.0) | 1 (4.0) | >0.999 |
| Questionnaires | | | | | |
| STOP-Bang questionnaire | 4.9 ± 1.4 | 4.7 ± 1.2 | 5.2 ± 1.3 | 5.5 ± 1.3 | 0.175 |
| Epworth sleepiness scale | 7.6 ± 5.0 | 6.9 ± 3.9 | 8.4 ± 5.2 | 6.4 ± 2.9 | 0.395 |
| Pittsburgh sleep quality index | 8.2 ± 3.8 | 7.5 ± 3.0 | 8.3 ± 4.2 | 7.6 ± 3.9 | 0.802 |
| Insomnia severity index | 9.1 ± 6.6 | 9.5 ± 5.0 | 9.6 ± 5.0 | 7.2 ± 4.6 | 0.384 |
| Beck depression inventory | 9.6 ± 9.1 | 8.3 ± 6.1 | 13.8 ± 8.2 | 13.4 ± 10.5 | 0.052 |
| Polysomnography | | | | | |
| AHI, /h | 21.9 ± 13.5 | 29.3 ± 15.0 | 40.2 ± 16.5 | 61.2 ± 20.9 | <0.001 |
| Supine AHI, /h | 37.0 ± 20.5 | 40.7 ± 19.8 | 57.5 ± 23.9 | 76.3 ± 26.0 | <0.001 |
| Nonsupine AHI, /h | 9.3 ± 15.0 | 12.1 ± 12.2 | 23.0 ± 19.6 | 44.0 ± 28.1 | <0.001 |
| REM AHI, /h | 24.1 ± 17.7 | 37.0 ± 17.8 | 39.7 ± 19.0 | 60.4 ± 22.1 | <0.001 |
| NREM AHI, /h | 20.1 ± 13.8 | 26.1 ± 17.2 | 36.6 ± 18.3 | 58.7 ± 21.9 | <0.001 |
| Isolated REM OSA^†^ | 1 (3.6) | 1 (3.7) | 2 (8.0) | 0 (0.0) | 0.642 |
| RDI, /h | 22.9 ± 14.4 | 29.7 ± 14.8 | 40.5 ± 16.6 | 61.2 ± 20.9 | <0.001 |
| Mean SpO_2_, % | 94.8 ± 1.3 | 92.9 ± 1.2 | 91.5 ± 1.1 | 86.4 ± 5.8 | <0.001 |
| Lowest SpO_2_, % | 86.1 ± 4.3 | 79.4 ± 7.1 | 76.0 ± 7.5 | 66.5 ± 11.7 | <0.001 |

Values are presented as mean ± SD or number (%).

^*^Heavy drinkers were defined as individuals who drank at least twice a week, and the average amount of alcohol consumed ≥7 drinks at a time for men and ≥ 5 drinks for women.

^†^Isolated REM OSA was defined as an overall AHI ≥5/h, a REM AHI/NREM AHI ratio ≥2, a NREM AHI <5/h, a REM AHI >5/h, and REM sleep ≥30 minutes.

Abbreviations: AHI, apnea–hypopnea index; ALT, alanine transaminase; AST, aspartate transaminase; BP, blood pressure; BMI, body mass index; BUN, blood urea nitrogen; COPD, chronic obstructive pulmonary disease; FEV_1_, forced expiratory volume in 1 s; FVC, forced vital capacity; HbA1c, glycated hemoglobin; HDL, high-density lipoprotein; LDL, low-density lipoprotein; NREM, nonrapid eye movement; OSA, obstructive sleep apnea; RDI, respiratory disturbance index; REM, rapid eye movement; SpO_2_, oxygen saturation; STOP-Bang questionnaire, Snoring, Tiredness, Observed apnea, high blood Pressure-Body mass index, Age, Neck circumference, and Gender questionnaire; T90, percent night time with oxygen saturation <90%; WBC, white blood cells.


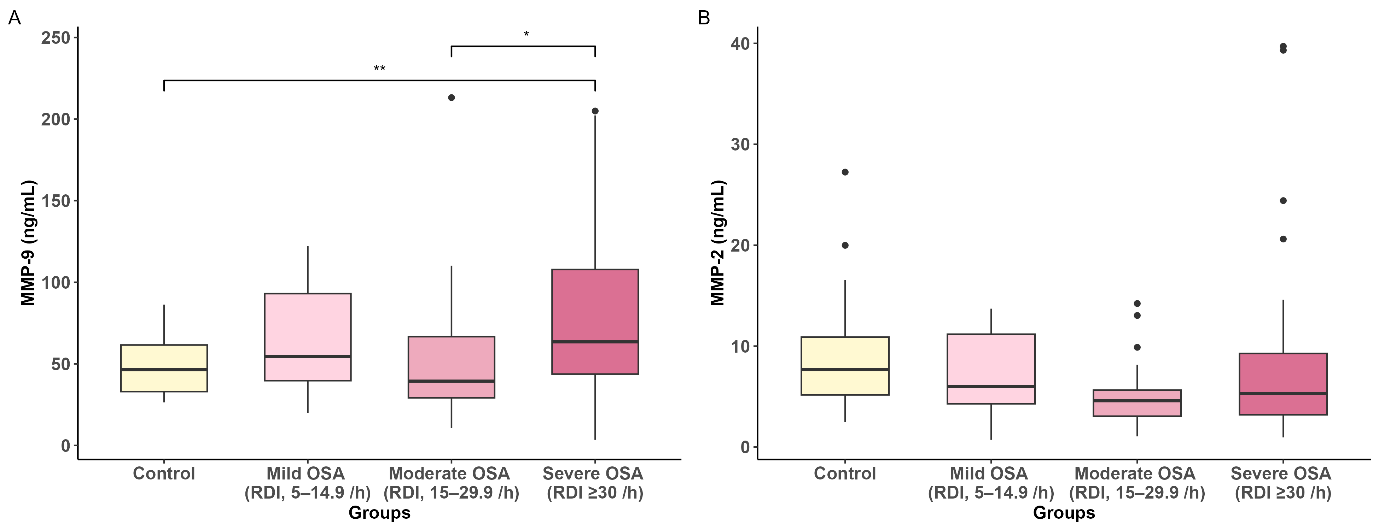


**FIGURE S1** Serum levels of MMP-9 (A) and MMP-2 (B) according to the RDI. The groups were compared by one-way analysis of variance followed by Tukey’s honest significance test. **p* < 0.05; ***p* < 0.01.

MMP-9, matrix metalloproteinase-9; OSA, obstructive sleep apnea; RDI, respiratory disturbance index.


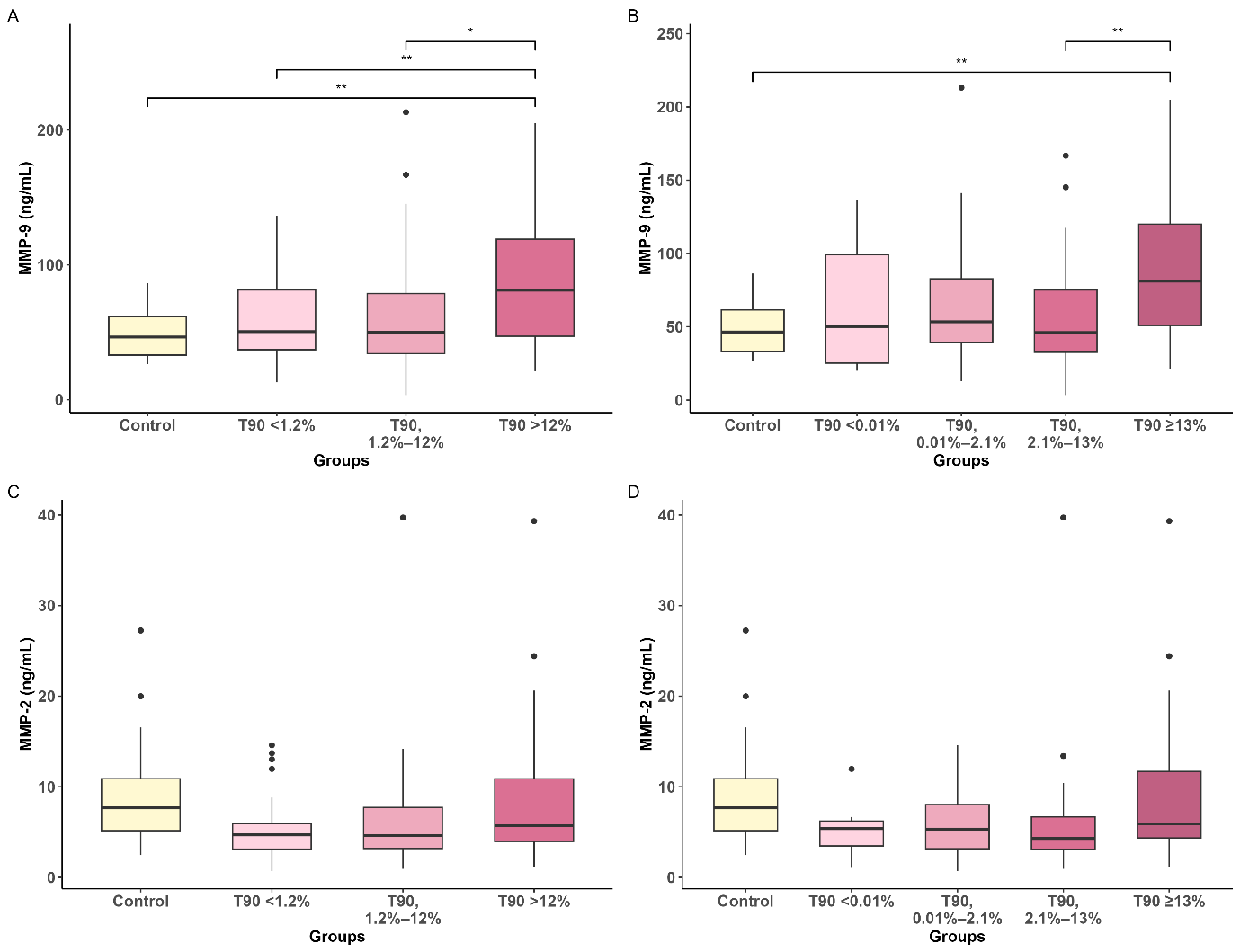


**FIGURE S2** Serum levels of MMP-9 (A, B) and MMP-2 (C, D) according to the different categories of T90. T90 is grouped into tertiles (<1.2%, 1.2%–12%, >12%), as suggested by Campos-Rodriguez et al. (A, C), and quartiles (<0.01%, 0.01%–2.1%, 2.1%–13%, ≥13%), as recommended by Justeau et al. The groups were compared by one-way analysis of variance followed by Tukey’s honest significance test. **p* < 0.05; ***p* < 0.01.

MMP, matrix metalloproteinase; T90, percent night time with oxygen saturation <90%.

Campos-Rodriguez, F., Martinez-Garcia, M. A., Martinez, M. et al. (2013). Association between obstructive sleep apnea and cancer incidence in a large multicenter Spanish cohort. *Am J Respir Crit Care Med*, **187**: 99–105.

Justeau, G., Gerves-Pinquie, C., Le Vaillant, M. et al. (2020). Association between nocturnal hypoxemia and cancer incidence in patients investigated for OSA: Data from a large multicenter French cohort. *Chest*, **158**: 2610–2620.


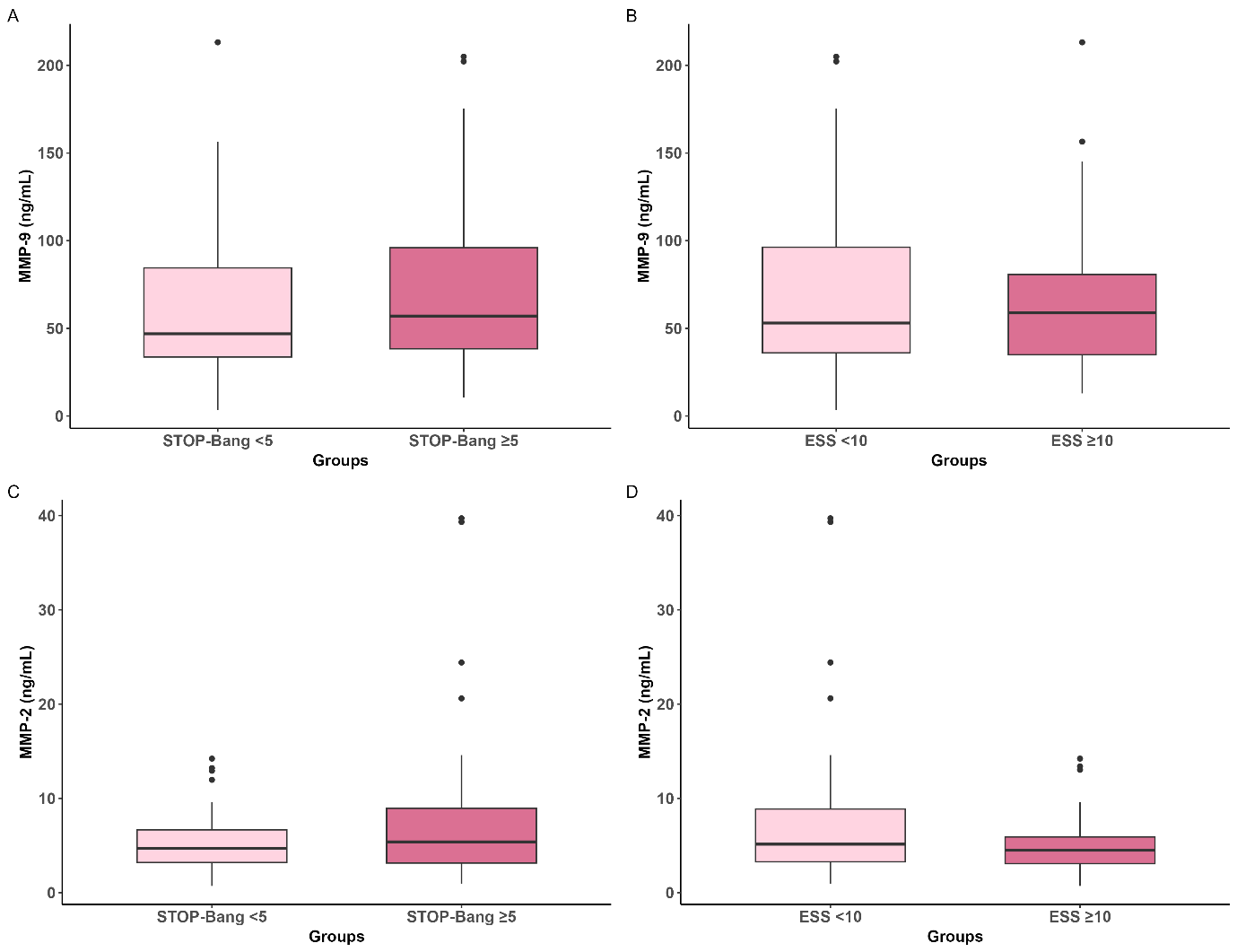


**FIGURE S3** Serum levels of MMP-9 and MMP-2 according to STOP-Bang questionnaire scores (<5 vs. ≥5; A and C) or ESS scores (<10 vs. ≥10; B and D) in patients with OSA.

ESS, Epworth sleepiness scale; MMP, matrix metalloproteinase; OSA, obstructive sleep apnea; STOP-Bang questionnaire, Snoring, Tiredness, Observed apnea, high blood Pressure-Body mass index, Age, Neck circumference, and Gender questionnaire.


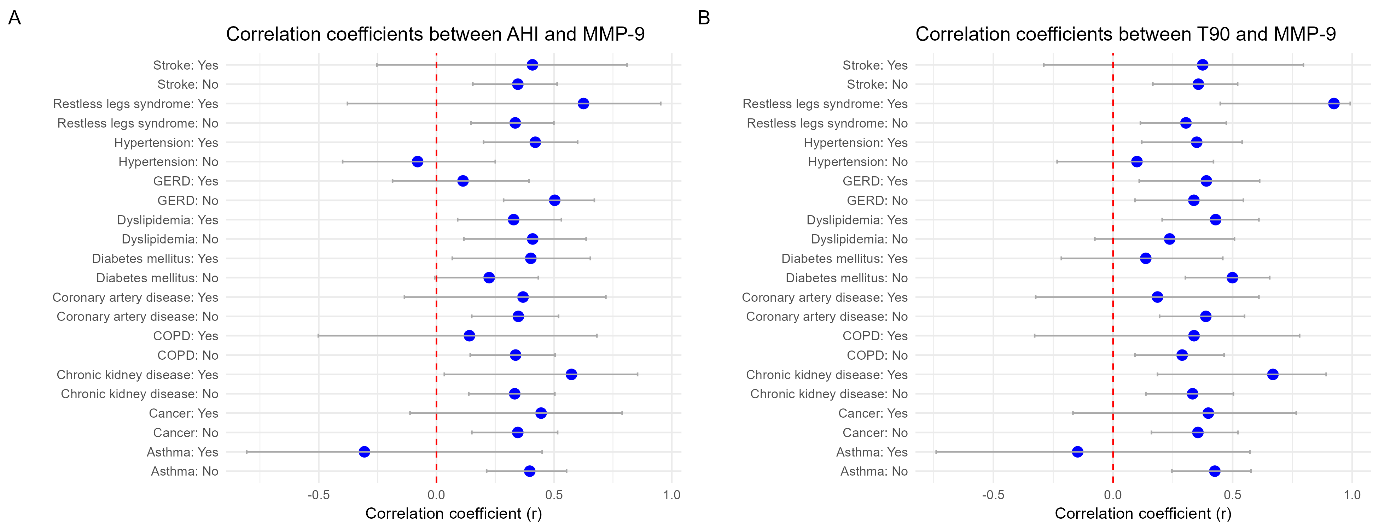


**FIGURE S4** Pearson correlation coefficients between AHI or T90 and MMP-9 across comorbidity subgroups in patients with OSA.

AHI, apnea–hypopnea index; COPD, chronic obstructive pulmonary disease; GERD, gastroesophageal reflux disease; MMP-9, matrix metalloproteinase-9; OSA, obstructive sleep apnea; T90, percent night time with oxygen saturation <90%.
